# Seasonality and pathogen transmission in pastoral cattle contact networks

**DOI:** 10.1098/rsos.170808

**Published:** 2017-12-06

**Authors:** Kimberly VanderWaal, Marie Gilbertson, Sharon Okanga, Brian F. Allan, Meggan E. Craft

**Affiliations:** 1 Department of Veterinary Population Medicine, University of Minnesota, St Paul, MN, USA; 2 Department of Entomology, University of Illinois Urbana-Champaign, Urbana, IL 61801, USA

**Keywords:** network analysis, infectious disease, animal movement, ecology, pathogen

## Abstract

Capturing heterogeneity in contact patterns in animal populations is essential for understanding the spread of infectious diseases. In contrast to other regions of the world in which livestock movement networks are integral to pathogen prevention and control policies, contact networks are understudied in pastoral regions of Africa due to the challenge of measuring contact among mobile herds of cattle whose movements are driven by access to resources. Furthermore, the extent to which seasonal changes in the distribution of water and resources impacts the structure of contact networks in cattle is uncertain. Contact networks may be more conducive to pathogen spread in the dry season due to congregation at limited water sources. Alternatively, less abundant forage may result in decreased pathogen transmission due to competitive avoidance among herds, as measured by reduced contact rates. Here, we use GPS technology to concurrently track 49 free-roaming cattle herds within a semi-arid region of Kenya, and use these data to characterize seasonal contact networks and model the spread of a highly infectious pathogen. This work provides the first empirical data on the local contact network structure of mobile herds based on quantifiable contact events. The contact network demonstrated high levels of interconnectivity. An increase in contacts near to water resources in the dry season resulted in networks with both higher contact rates and higher potential for pathogen spread than in the wet season. Simulated disease outbreaks were also larger in the dry season. Results support the hypothesis that limited water resources enhance connectivity and transmission within contact networks, as opposed to reducing connectivity as a result of competitive avoidance. These results cast light on the impact of seasonal heterogeneity in resource availability on predicting pathogen transmission dynamics, which has implications for other free-ranging wild and domestic populations.

## 1. Introduction

Patterns of contact within populations have important implications for the spread of pathogens, and are driven by interactions between population density, resource distribution, animal movements, and human and animal behaviour. Populations rarely mix homogeneously and incorporating heterogeneity in contact patterns in models of pathogen transmission is critical for understanding and predicting the spread of infectious diseases [1–4]. For example, many livestock and human diseases, including measles, SARS and foot-and-mouth disease (FMD), have been shown to exhibit transmission networks with extreme positive skew, whereby a minority of cases are responsible for the majority of transmission events [2,5,6]. The ability to identify these key transmitters, or super-spreaders, is instrumental to efficient and effective control policies [6,7].

Driven in part by several high-profile outbreaks of FMD, bovine spongiform encephalopathy and other livestock pathogens in Europe and South America, many countries now implement animal traceability programmes to track the shipments of animals between farms [8–10]. Contact networks based on animal shipments between farms have proven key to understanding the transmission dynamics of past outbreaks, identifying high-risk herds, and optimizing future targeted surveillance and control measures [2,11–13]. However, many of the policy-informing insights gained from network models of pathogen spread in livestock are not yet available in systems where contact among herds is dictated by ecologically driven patterns of range use rather than shipments of animals between farms. Contact patterns in such systems [14], and within livestock industries in developing countries more generally [15], have rarely been studied, creating a need to better understand contact patterns among nomadic or pastoralist herds. Indeed, rangelands where livestock grazing is the primary land use account for approximately 45% of the Earth's surface, excluding Antarctica, and the majority of these areas lie within developing countries [16]. In Kenya, the arid and semi-arid rangelands where pastoralism occurs comprise 20% of the country's land area [17], and at least 60% of beef consumed in Kenya is produced in these regions [18]. Areas where pastoralism dominates are highly susceptible to outbreaks of infectious diseases, such as FMD, due to high levels of animal mobility and limited veterinary infrastructure [19–23], highlighting the importance of understanding pathogen transmission in pastoralist settings for economic and food stability.

For pastoralists in Kenya and elsewhere, animal movement is a primary response to spatial heterogeneity in resource and water availability, driven by seasonal and climatic variability, and also by drought [24]; mobility is necessary for maintaining sustainable livestock production in semi-arid landscapes [25]. In general, livestock movements can be summarized at scales ranging from transboundary imports and exports of animals, to highly local scales that focus on short-distance trade interactions or direct contact among herds in the context of daily foraging. In between these two extremes lie national supply chains and seasonal patterns of animal movement. Each of these scales has important implications for the spread and control of infectious diseases. For regions with pastoralist production systems [14,20], an understanding of the contact structure among mobile herds of livestock will help overcome challenges in applying appropriate spatial risk-based surveillance and disease control, including defining appropriate vaccination and control strategies for outbreaks of diseases such as FMD [26].

Previous efforts to quantify contact patterns in pastoralist herds include ego-based approaches [22], wherein data are collected on the frequency of contacts with other herds, but no data are collected on the identity of contacted herds. For example, in areas of Cameroon where transhumance occurs (i.e. herds have a definitive place of residence, but routinely move to other areas for grazing for extended periods of time), herds typically encounter up to 10 other herds per day [22]. To approximate who is in contact with whom, other authors have used sharing of watering points and grazing areas or overlap in home ranges as proxies of contact [14,20,27]. However, spatial proxies for between-herd contact may not adequately capture patterns of interaction pertinent to infectious disease transmission in pastoralist systems, such as the frequency and duration of contact.

In addition, seasonal shifts in precipitation dictate the availability of water and forage in semi-arid ecosystems, which ultimately changes the way animals use the landscape [28]. Given the importance of water in driving pastoral cattle grazing patterns, using pastoral cattle as a study system allows us to explore how seasonally scarce water resources impact contact patterns among grazing animals. Densities of pastoral cattle are often proportional to available resources [29], and ungulates typically decrease habitat overlap during the dry season due to increased competition for forage [30,31]. However, limited water availability may increase contact rates [14]. These seasonally driven changes in behaviour have the potential to affect the structure of contact networks, and thereby profoundly alter the dynamics of pathogen transmission through an ecosystem.

In this study, we describe the contact networks of cattle managed in a style similar to pastoralism, comparing the seasonal differences of these networks across wet and dry seasons. We also analyse the role of water and overnight stockades, or *bomas*, in driving patterns of contact. Finally, we simulate the spread of a highly infectious, FMD-like pathogen on these seasonal contact networks to assess any impact of seasonal differences in contact network structure on pathogen transmission between herds. We expect that contact networks will be more conducive to pathogen spread in the dry season due to congregation at water sources. Alternatively, less abundant forage may result in herds becoming dispersed across the landscape, thereby decreasing contact rates and opportunities for pathogen transmission. We test these hypotheses in a semi-arid region of central Kenya.

## 2. Methods

### 2.1. Study population

This study was conducted at Ol Pejeta Conservancy (OPC), a 364 km^2^ semi-arid savannah woodland ecosystem located in Laikipia county, Kenya (0° N, 36.7° E), from February to June, 2015. Laikipia county—where the primary land use is livestock husbandry—is characterized by distinct wet and dry seasons. OPC receives on average 900 mm of rainfall per year [32], with peak rainfall occurring in late-March–April and October–November. OPC is a commercial cattle ranch and wildlife conservancy, and cattle are minimally managed in a manner similar to pastoral communities within the county. Cattle are stockaded at night in *bomas* (i.e. corrals) and herded to water points and to graze in open rangeland each day. Boma locations are generally moved every few weeks to months, with boma and grazing locations selected by ranch managers and herders based upon resource availability. In general, cattle reared on commercial ranches have better access to water, forage and veterinary care than cattle kept on communal lands; in this study, cattle typically obtained water from permanent water troughs as opposed to drinking from rivers or natural water holes. Because of more consistent access to forage and water, commercial cattle are not involved in transhumance, and do not move long distances between grazing areas seasonally. However, the daily movement patterns of cattle within OPC resemble that of community cattle, leaving from central boma locations in search of available water and forage during the day, and returning at night. The density of herds on OPC was approximately 0.11 herds km^−2^, and average stocking density at the time of the study was 16.5 tropical livestock units (TLU) km^−2^. OPC is enclosed by a perimeter fence except for a few wildlife corridors, preventing contact with external herds.

### 2.2. Data collection

Forty-nine cattle herds within OPC were monitored via GPS technology during dry and wet periods in 2015. Although one GPS unit per herd was expected to be representative of the herd's movement [33], we tracked movement of each herd using two GPS units (i-gotU, model GT-600): one GPS unit attached by collar to a cow and the other carried by a human herder. GPS units recorded GPS coordinate locations, or ‘fixes’, every 15 min. Occasionally, a GPS unit would malfunction or be lost. Due to these circumstances, data were not available at times for some herds. Additionally, a GPS unit occasionally recorded a fix that would require unreasonably fast movement at a pace greater than 20 km h^−1^ between consecutive fixes. These fixes were considered erroneous and were eliminated from analysis. A total of 41 herds were monitored during the dry season, which occurred between 3 February and 30 March 2015, and a total of 42 herds were monitored during the wet season between 14 April and 15 June 2015. Because of the combining and splitting of herds over time, not all 49 herds existed in both seasons.

**Figure 1.**
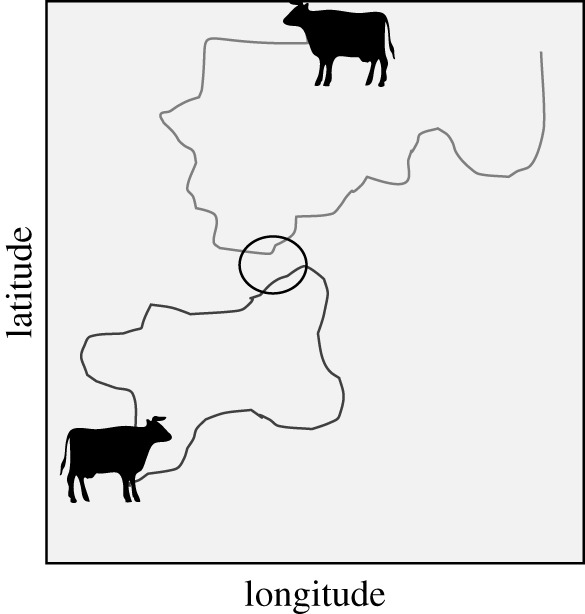
Schematic used to define contact from daily movements of two herds. Circled area indicates the location of a contact (defined as two GPS fixes from different individuals with locations less than 100 m apart within 60 min).

Contact between two herds was defined as the temporally closest pair of fixes that occurred within 100 m and 1 h of each other (figure 1). Research on pastoralist herds elsewhere indicates that the average distance between animals of the same herd is approximately 50 m [33,34]. Thus, our spatio-temporal window defining a contact was chosen as a reasonable representation of contact between animals in different herds in this system. Wet and dry season data were assessed separately. This resulted in dry and wet season networks that were based on similar numbers of days in the study (48 versus 43 days for the dry and wet season, respectively) and total numbers of GPS fixes (37 826 and 37 031 fixes in the dry and wet season, respectively), thereby minimizing the effect of any differences in sampling effort between the two seasons.

A weighted contact network was created from each of the wet and dry season data. Not every herd was monitored for every day of the study. Therefore, we calculated a standardized association index for each pair of herds, defined as the number of days in which they were in contact divided by the total number of days for which data were available for those two herds. Links in the network were weighted according to the value of this index. Additionally, an unweighted dynamic network, which included the temporal sequence and locations of between-herd contacts, was constructed for spatial analyses of contacts and modelling the spread of infectious disease. Contact locations were defined as the midpoint between the two GPS fixes involved in each contact. Because a single fix location for herd A could be considered in contact with several fix locations for herd B, only the first contact per hour for each dyad was used to define the spatial location.

### 2.3. Network analysis

We quantified the centrality and connectivity of individual herds in the dry and wet season contact networks using herd-level metrics of degree, tie-strength, closeness and betweenness. Degree was defined as the number of different herds in contact with a herd over the course of the season, standardized by the number of days for which data was available for the herd. Tie-strength was defined as the herd's mean number of contacts per day. Closeness quantifies the extent to which a herd is located centrally in the network, and is calculated as the harmonic mean of the weighted path length between the focal herd and all others [35]. Betweenness quantifies the extent to which individual herds occupy positions in the network that are important for mediating connections between others, and is calculated based on the number of paths that pass through the focal node when the shortest weighted paths are traced between all other pairs of herds [35]. Weighted versions of both closeness and betweenness were used. Closeness and betweenness metrics were normalized by dividing by (*n* *− *1), where *n* is the number of herds in the network [35]. Tests for significant differences between the herd-level metrics for each network were completed by ANOVA with permutated *p*-values using the *aovp* function in the R package *lmPerm* [36]. In addition, two-sided *t*-tests with permutated *p*-values, performed using the *permTS* function in the R package *perm* [37], were used to test for significant differences in degree between the dry and wet season. In all cases, 1000 permutations were used to calculate *p*-values. We also tested for correlations between herd-level metrics and the number of days each herd was in the study using a two-sided Spearman's *rho* statistic.

Network-level metrics included density and centralization. Density is calculated as the number of links that occur in the network divided by the total number possible if all herds were connected to one another, and provides a metric of the overall connectivity of the network [35]. Values range from 0 (completely unconnected) to 1 (every herd is connected to all others). Degree centralization, closeness centralization and betweenness centralization each quantify the extent to which a minority of nodes are critical for maintaining overall connectivity in the network. High levels of centralization are often considered to be indicative of the presence of potential super-spreaders, or well-connected nodes that are responsible for a majority of pathogen transmission within the network. In addition, we examined the network for community structure, or groups of nodes that tend to interact with one another more frequently than with nodes outside the community. Community structure is expected to slow the spread of pathogens, particularly when the strength of community structure is high [38–40]. The number of communities within a network was calculated with a 7-step walktrap community-finding algorithm [41]. The strength of community structure was measured with modularity, which can be interpreted as the insularity of communities within a population. Modularity is calculated as the relative frequency of within- versus between-community interactions, with larger values indicating stronger community structure [42]. Networks with moderate to strong community structure will have modularity values ranging from 0.3 to 0.7 [42]. All network analyses were performed using the *igraph* package in R [41].

### 2.4. Proximity analysis

Proximity of contact locations to bomas, water troughs and spray races (i.e. acaricide application facilities for tick control) were compared to investigate these locations as potential drivers of contact. Bomas were located by identifying the most common overnight locations for each herd. Coordinates of water troughs and spray races were provided by OPC. To assess whether between-herd contacts were located closer to points of interest (e.g. bomas) than expected by chance, a sample of 1000 random locations was taken from all GPS fixes during the study period. We then calculated the distance from each contact and random location to the nearest boma, water trough and spray race. Two-sided *t*-tests with permutated *p*-values were used to test for significant differences between the distances from contact and random locations to bomas, water troughs and spray races. In addition, two-sided *t*-tests with permutated *p*-values were used to test for significant differences between the wet and dry season results.

To evaluate whether competitive avoidance occurred in the dry season, we examined seasonal differences in ‘on-forage’ contact rates, where on-forage was defined as contacts that were located at least 500 m away from bomas, water troughs and spray races. We repeated our analysis of degree and tie-strength for networks based only on these contact occurrences. To assess the sensitivity of results to the definition of on-forage contact, we replicated the analysis with the threshold of on-forage contact set to 200 or 1000 m from bomas, water troughs and spray races.

### 2.5. Pathogen transmission model

We used a stochastic, compartmental model to investigate the impact of seasonal differences in contact patterns on the spread of an acute, highly infectious pathogen loosely resembling FMD (a pathogen with negligible mortality and high within-herd morbidity). In this epidemiological model, the herd was considered the epidemiological unit, given that rapidly spreading pathogens such as FMD and other viruses are likely to infect a large proportion of a herd within a short time period [43]. In this SEI modelling approach [44], each herd in the network was defined as either Susceptible, Exposed, or Infected, and herds transitioned between compartments based on a set of probabilities. The probability that a susceptible herd will become exposed during a specific time step in the model is based on the probability that transmission occurs as a result of contact with infected herds:

$$P({\text{herd}}_{j}\text{ is exposed in time }t)=1-{\left(1-\mu \right)}^{k_{t}},$$

where *μ* is the probability of between-herd transmission given a contact, and *k_t_* is the number of infectious contacts herd *j* experienced on day *t* in the dynamic contact network. Exposed herds transition to Infected with rate 1/*λ*, where *λ* represents the period of the time until the herd is infectious (here, defined as 2 days). A recovered stage was not modelled given the short duration of the available network data (56 days); in a study of within-herd dynamics of FMD, for example, new clinical cases were reported up to 35 days after the initial case [45]. Simulations were run using either the wet season or dry season contact networks and seeded in a single, randomly selected index herd, which was meant to emulate a single spillover event from wildlife or another external source of introduction, and subsequent spread in cattle. Direct contact between cattle and wildlife species in this system is rare [46], and therefore wildlife species were not included in the model. Thus, following the initial introduction event, within-species contact was assumed to dominate pathogen transmission for a highly contagious, acute pathogen such as FMD. The model was run for 56 days for the wet and dry season simulations. 1000 simulations were performed for both the wet season and dry season with either lower (*μ* = 0.5) or higher (*μ* = 0.75) probabilities of transmission given contact. Model output was summarized as the proportion of herds that became infected, and differences between seasons were compared using *t*-tests.

## 3. Results

**Figure 2.**
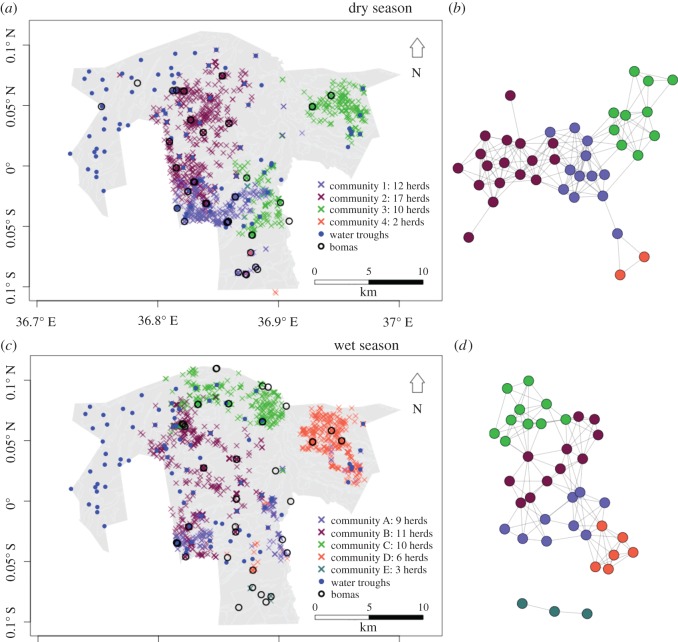
Map of locations where contacts occurred between herds during the dry (*a*) and wet (*c*) seasons, with colours of ‘x's’ indicating community identity. Between-herd network of cattle herds in the dry (*b*) and wet (*d*) seasons, with circles indicating individual herds and colours indicating community identity of the herd.

**Figure 3.**
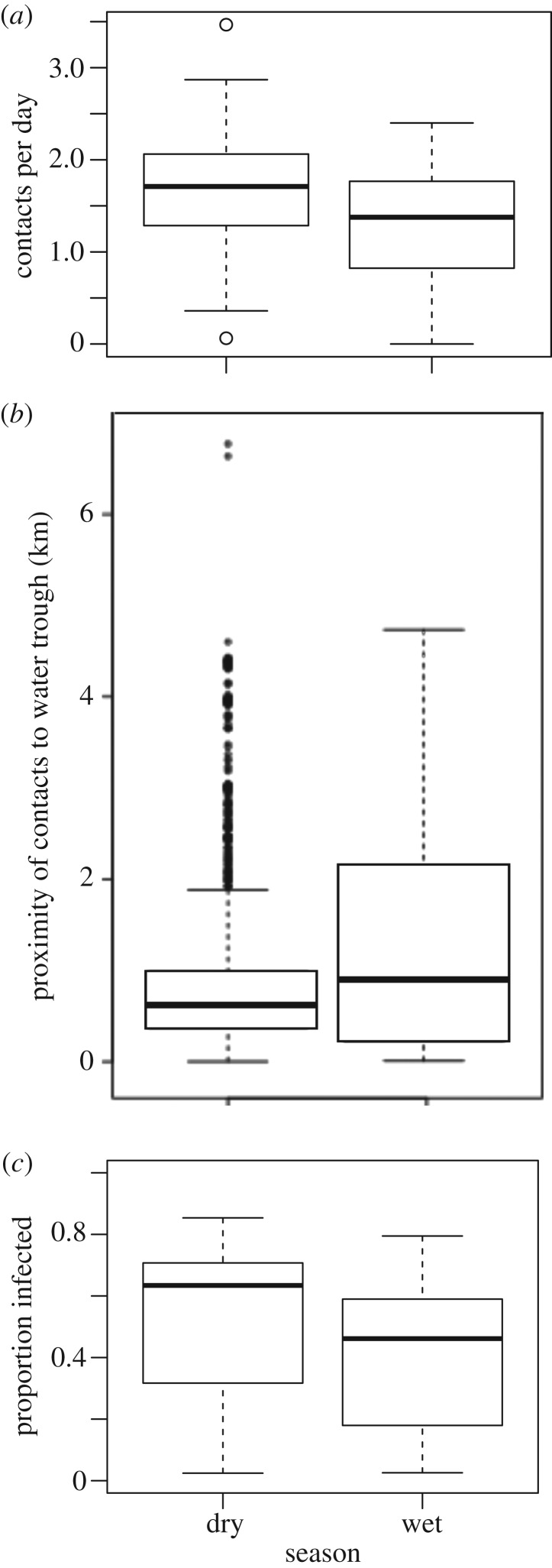
(*a*) Contacts per day, (*b*) proximity of contacts to water sources, and (*c*) proportion of herds infected (*μ* = 0.75) in simulated outbreaks in the dry and wet season networks.

Overall, our sampling approach yielded 17 425 and 18 136 GPS fixes that met our definition of contact in the dry and wet season, respectively, though many of these were between herds which were in close proximity for multiple consecutive fixes during a day. When counting each unique herd-to-herd contact only once per day, 682 contacts occurred out of 847 herd-days in the dry season and 633 contacts out of 892 herd-days in the wet season. 117 on-forage contacts (i.e. contact between herds that occurred greater than 500 m from bomas, water troughs and spray races) occurred in the dry season, and 139 occurred in the wet season. The network of between-herd contacts was highly connected, with approximately 10% of connections occurring relative to the total number possible in a completely connected network (density of 0.10 and 0.07 in the dry and wet season, respectively, figures 2 and 3). In the dry season, all herds had contacts with another herd, while in the wet season three herds were disconnected from the main network. Centralization scores for closeness and degree indicated moderate amounts of centralization of contacts around a core subset of influential nodes. Degree centralization was 0.17 and 0.15 and closeness centralization was 0.24 and 0.07 in the dry and wet seasons, respectively. Seasonal differences were more marked for closeness centralization, with the dry season exhibiting higher levels of centralization.

On average, cattle herds contacted other herds at a rate of 1.65 herds per day (range: 0.06–3.46) during the dry season, and 1.36 contacts per day (range: 0–2.4) during the wet season (*p* = 0.021, Kruskal–Wallis test, figure 3*a*). Herds made on average 0.27 on-forage contacts per day (range: 0–1.05) in the dry season and 0.26 on-forage contacts per day (range: 0–1.16) in the wet season. This difference was not significant (*p* = 0.14, Kruskal–Wallis test). Contacts per day were correlated with days in study in the wet season (*ρ* = 0.46, *p* = 0.002), but not the dry (*ρ* = −0.16, *p* > 0.33). However, the significant correlation in the wet season between contacts per day and days in study disappears if we restrict the analysis to only those herds that were in the study for at least 8 days. Only two herds failed to meet this criterion in the dry season.

The median number of unique contacts made by each herd was 9 (range: 1–15) and 6 (range: 1–12) in the dry and wet seasons respectively. When standardized by days in the study, the number of unique contacts was 0.5 contacts per day (range: 0.06–1.5) during the dry season, and 0.3 contacts per day (range: 0–1.2) during the wet season (*p* = 0.007, Kruskal–Wallis test). The number of unique contacts was significantly correlated with days in study, even after standardization (*ρ* = −0.49, *p* = 0.001). This negative correlation suggests that the number of new, unique contacts stagnates over time, most likely due to the fact that a herd eventually encounters all local neighbouring herds and would need to move to a new location to encounter different herds.

The community analysis revealed that herds within OPC could be divided into four and five communities in the dry and wet season, respectively (figure 2). We used numbers in the dry season and letters in the wet season to indicate community identities to emphasize that the communities are composed of different herds each season. Maps of contact locations reveal a clear correlation between geographical location and community membership (figure 2). The modularity of community structure in the cattle networks was 0.33 and 0.40 in the dry and wet season, respectively, indicating that the strength of the community structuring within the network was only moderate.

In the dry season, contact locations were significantly closer to bomas, water troughs and spray races than random cattle locations (*p* < 0.001 in all cases). In the wet season, however, contacts were closer to bomas and spray races than random (*p* < 0.001 for both cases), but not to water troughs (*p* = 0.54). When comparing across seasons, contacts were significantly closer to water troughs (figure 3*b*) and bomas during the dry season than during the wet season (*p* < 0.001), but there was no significant seasonal difference for spray races (*p* = 0.47).

**Figure 4.**
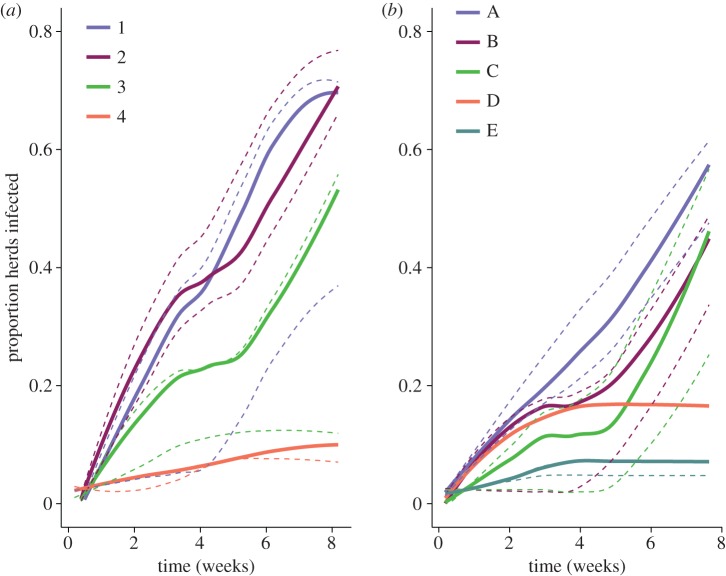
Epidemic curve of the proportion of herds infected over time in the (*a*) dry and (*b*) wet season, with colours indicating the community of the initially infected herd. Solid lines indicate medians and dotted lines indicate the interquartile ranges.

Our model demonstrated that pathogens spread widely in both seasonal networks and infected a high proportion of herds. When the probability of infection given contact (*μ*) was 0.5, simulated pathogen outbreaks in the dry and wet seasons infected a median of 49% (interquartile range (IQR): 27–66%) and 35% (IQR: 17–43%) of herds, respectively. On average, 10% more herds became infected in the dry season than the wet season. When the probability of infection given contact was 0.75, simulated pathogen outbreaks in the dry and wet seasons infected a median of 63% (IQR: 32–71%) and 44% (IQR: 17–56%) of herds in the simulated outbreaks, respectively (*p* < 0.001, figures 3*c* and 4). In most cases, new herds were still becoming infected at the end of the simulation, suggesting that outbreaks would continue to persist for more than the 56 days for which dynamic contact network data were available (figure 4). The size of epidemics was also strongly influenced by the identity of the community in which the initially infected index herd was located (figure 4).

## 4. Discussion

The network of contacts among mobile cattle herds in central Kenya demonstrated high levels of connectivity, with herds typically coming into close proximity to other herds 1–2 times per day. Water was a significant driver of between-herd contacts in the dry season, but not the wet season, probably reflecting more frequent use of water sources during dry periods of the year. This clustering of contacts around water resources resulted in networks with both higher contact rates (figure 3*a*) and higher potential for pathogen super-spreading, as measured by centralization of herd-level connectivity scores that were up to 3.5 times higher in the dry season. Consistent with expectations given network structure, simulated outbreaks were larger in the dry season than in the wet season. Results support the hypothesis that congregation around limited water sources enhances connectivity and transmission within contact networks, and may result in the presence of super-spreading herds within the population. At the same time, we do not find evidence that competition over limited forage resources during the dry season leads to competitive avoidance among herds, as there was no difference in on-forage contact rates across seasons.

Our simulation model demonstrated that even in the absence of other seasonally induced factors which could affect pathogen transmission, the predicted size of outbreaks was influenced by seasonal differences in contact structure alone, with larger outbreaks occurring during the dry season. Other factors, including poor body condition during the dry season or changes in pathogen transmissibility in different weather conditions, may further alter predictions of outbreak size, but differences emerge even without considering such factors. Thus, ignoring seasonal changes in network structure in disease transmission models may alter epidemiological predictions and reduce our ability to understand and predict the spread of infectious diseases. A potential application of the observed seasonal differences in contact networks for directly transmitted pathogens is in vaccination policy. FMD vaccination, for example, is only protective against infection for a relatively short period and herd turnover creates a constant influx of new susceptible individuals. Repeated vaccinations (sometimes 2–3 times per year) are required to maintain immunity in a population [45,47]. If frequent vaccination cannot be maintained, our results suggest that vaccination at the beginning of dry periods could have the maximum impact in limiting pathogen transmission.

The results of our network analysis enhance baseline knowledge on contact patterns among cattle herds in pastoralist production systems, and are useful for modelling the dynamics of pathogen spread. However, the reported contact patterns are potentially density dependent, and caution should be used in extrapolating these contact rates to other pastoralist systems. Indeed, findings elsewhere report that the number of animals in an area is proportional to primary productivity [29], and higher densities of herds would probably translate into higher likelihoods of contact [44]. In addition, herd densities within OPC did not vary with seasonal changes in forage abundance as might be expected in communal rangelands. Long-distance connectivity patterns over extended time scales based on seasonal movements also were not captured here. Consequently, the results of this study should be interpreted within the context of this scale, i.e. contacts within a local area within a single season. Understanding regional networks accounting for seasonal movements and longer timespans would facilitate a more holistic picture of cattle movements and contact patterns in this part of Kenya [27].

Approximating herd-to-herd contact using presumed overlap in daily grazing areas or home ranges, as has been done in other studies, may overestimate contacts. For example, a 5 km buffer around overnight stockade locations was used to approximate a herd's space use in Cameroon [27] and neighbouring herds that overlapped in presumed space use were considered homogeneously mixed. Here, had we used the average daily distance moved from the boma to approximate a grazing radius (2–5 km), we would have overestimated the number of daily contacts made by herds compared to the estimates generated from daily movement patterns using GPS. It may be possible that such an approximation would be fairly reasonable if contacts were summarized on a weekly or monthly scale. In this case, a herd may contact all herds within its area at least once. However, this approach would obscure the temporal sequence of contacts, which can substantially alter predictions of disease spread [48].

The results presented here assume that transmission only occurs through direct contact among herds and not through contaminated forage or water. Such environmental transmission probably would increase transmission between herds [49]. We also assumed that, during an outbreak, within-species transmission is substantially more important than between-species, and thus ignored the presence of wildlife species in this system. We justify this assumption because contact between species is far less frequent than within species [46], and cattle and wildlife appear to host genetically distinct strains of FMD in Kenya, which further suggests that contact may be rare [50]. However, we cannot discount the possibility that other ungulate species may be involved in disease outbreaks. Thus, more research is required to parametrize a multi-species model, including data on pathogen persistence in the environment, patterns of contact among species and the frequency of interspecific transmission.

The use of networks in developing disease surveillance and control strategies has led to a productive interface between research and policy during the past 20 years [51–54]. Unfortunately, these methods are generally not available in regions characterized by pastoral or nomadic livestock husbandry practices, such as many areas of Africa. Moreover, existing methods for livestock network construction are based on shipments of animals between geographically fixed livestock premises, and are inappropriate for pastoral and nomadic systems. We also demonstrate that ecological drivers of movement are critical for understanding contact patterns, though more work is needed across a range of landscapes to develop a more complete understanding of how seasonal variation and landscape heterogeneity impacts contact patterns. This research represents one of the first reports of patterns of between-herd contact in pastoralist systems, which is a key first step for understanding the dynamic nature of pathogen spread in these systems.

## Data accessibility

Data and modelling code are deposited in the Data Repository for the University of Minnesota (DRUM: https://doi.org/10.13020/D60D42).
